# The Easy Part of the Hard Problem: A Resonance Theory of Consciousness

**DOI:** 10.3389/fnhum.2019.00378

**Published:** 2019-10-31

**Authors:** Tam Hunt, Jonathan W. Schooler

**Affiliations:** Psychological and Brain Sciences, University of California, Santa Barbara, Santa Barbara, CA, United States

**Keywords:** consciousness, Hard Problem of consciousness, resonance, self-organization, coherence

## Abstract

Synchronization, harmonization, vibrations, or simply *resonance* in its most general sense seems to have an integral relationship with consciousness itself. One of the possible “neural correlates of consciousness” in mammalian brains is a specific combination of gamma, beta and theta electrical synchrony. More broadly, we see similar kinds of resonance patterns in living and non-living structures of many types. What clues can resonance provide about the nature of consciousness more generally? This paper provides an overview of resonating structures in the fields of neuroscience, biology and physics and offers a possible solution to what we see as the “easy part” of the “Hard Problem” of consciousness, which is generally known as the “combination problem.” The combination problem asks: how do micro-conscious entities combine into a higher-level macro-consciousness? The proposed solution in the context of mammalian consciousness suggests that a shared resonance is what allows different parts of the brain to achieve a phase transition in the speed and bandwidth of information flows between the constituent parts. This phase transition allows for richer varieties of consciousness to arise, with the character and content of that consciousness in each moment determined by the particular set of constituent neurons. We also offer more general insights into the ontology of consciousness and suggest that consciousness manifests as a continuum of increasing richness in all physical processes, distinguishing our view from emergentist materialism. We refer to this approach, a meta-synthesis, as a (general) resonance theory of consciousness. We offer some suggestions for testing the theory.

At the heart of the universe is a steady, insistent beat: the sound of cycles in sync…. [T]hese feats of synchrony occur spontaneously, almost as if nature has an eerie yearning for order.

Steven Strogatz, *Sync: How Order Emerges From Chaos in the Universe, Nature and Daily Life* (2003)

If you want to find the secrets of the universe, think in terms of energy, frequency and vibration.

Nikola Tesla (1942)

## Introduction

### Distinguishing the “Easy Part” and the “Hard Part” of the Hard Problem of Consciousness

The Hard Problem of consciousness refers to the vexing challenge of understanding how matter (e.g., the human brain) is capable of having subjective experience (Chalmers, 1996; Goff, 2017) – what has historically been known as the mind/body problem. Is there an “easy part” and a “hard part” to the Hard Problem of consciousness? In this paper, we suggest that there is. The harder part is arriving at a philosophical position with respect to the relationship of matter and mind. This paper, a meta-synthesis, is about the “easy part” of the Hard Problem – the specific mechanisms of consciousness in physical structures. We address the “hard part” of the Hard Problem briefly in this introduction.

Our argument in this paper, in quick summary, is as follows: (1) All things resonate in some manner; (2) in many circumstances, things resonating in proximity will start resonating together at the same frequency, achieving a shared resonance; (3) we take panpsychism, the notion that all matter is associated with at least some degree of mind/subjectivity/consciousness, as our metaphysical starting point and don’t dwell long on why we have arrived at this position since that debate is addressed elsewhere; (4) achieving a shared resonance is what leads micro-conscious entities to combine into macro-conscious entities, often with a phase transition in the speed of information sharing resulting from that shared resonance.

The notion of resonance (also known as synchrony, coherence, or shared vibrations) has a long history in neuroscience. Crick and Koch feature this concept prominently in their neurobiological theory of consciousness (Crick and Koch, 1990; Koch, 2004). Fries similarly identifies the process of “communication through coherence” (neuronal synchrony/resonance) as a critical component of neural function (Fries, 2005, 2015). Dehaene, in his Global Workspace Theory, highlights the role of long-range synchrony between cortical areas as a key “signature of consciousness” (Koch, 2004; Dehaene, 2014). Grossberg (2017) introduced an Adaptive Resonance Theory (ART) that argues that “all conscious states are resonant states,” but that not all resonant states are conscious states. Freeman and Vitiello rely on resonance and phase transitions in their approach to explaining brain dynamics (Freeman and Vitiello, 2006). Pockett has proposed an electromagnetic field theory of consciousness that relies on “synchronization during the feedback of activity” to distinguish conscious from non-conscious fields (Pockett, 2000, 2012). As a final recent example, the concept of resonance is central to Bandyopadhyay’s (2019) Fractal Information Theory of consciousness (Sahu et al., 2013a, b; Singh et al., 2018).

We build upon this extensive body of work in developing our general resonance theory of consciousness. We take panpsychism (also known as panexperientialism) as our metaphysical starting point. This philosophical stance suggests that all matter has at least some associated mind/experience and vice versa, albeit highly rudimentary in the large majority of instances. All things and processes have both mental and physical aspects.^1^ Matter and mind are two sides of the same coin.

Panpsychism is one of many possible approaches that addresses the “hard part” of the Hard Problem. We adopt this position for the reasons described in Section “The “Hard Part” of the Hard Problem” below, which we and authors have discussed in more depth elsewhere (Chalmers, 1996; Griffin, 1998; Hunt, 2011; Goff, 2017). This first step is particularly powerful if we adopt a Whiteheadian version of panpsychism (Whitehead et al., 1929; Griffin, 1998).

Reaching a position on this fundamental question of how mind relates to matter must be based on a “weight of plausibility” approach, rather than on definitive evidence, because establishing definitive evidence with respect to the presence of mind/experience is a difficult problem. We must generally rely on examining various “measurable correlates of consciousness” in judging whether entities other than ourselves are conscious – even with respect to other humans – since the only consciousness we can know with certainty is our own.

We propose below some methods, however, for testing our proposed approach and an in-progress paper (Hunt, 2019) fleshes out these suggestions, relying on examination of neural correlates of consciousness, behavioral correlates of consciousness, and creative correlates of consciousness, which we view as various types of the broader category of “measurable correlates of consciousness.”

Positing panpsychism avoids the problems of emergence because under this approach consciousness doesn’t emerge. Consciousness is, rather, always present, at some level, even in the simplest of processes, but it “complexifies” as matter complexifies, and vice versa. Consciousness starts very simple and becomes more complex and rich under the right conditions, which in our proposed framework rely on resonance mechanisms. Biologically evolved entities rely on resonance between their constituent parts to achieve far more complex types of consciousness. In our version of panpsychism, neither matter nor mind is primary; they are coequal.

We acknowledge the challenges of adopting this perspective, but encourage readers to consider the many compelling reasons offered for this view that are reviewed elsewhere (Chalmers, 1996; Griffin, 1998; Skrbina, 2005; Strawson and Freeman, 2006; Hunt, 2011; Schooler et al., 2011; Schooler, 2015; Goff, 2017).

### The Combination Problem

Taking a position on the overarching ontology is the first (and arguably hardest) step in addressing the Hard Problem. But this leads to the related questions: at what level of organization does consciousness reside in any particular process? Is an atom conscious? A chair? An ant? A bacterium? Or are only the smaller constituents, such as atoms or molecules, of these entities conscious? And if there is some degree of consciousness even in atoms and molecules, as panpsychism suggests (albeit of a very rudimentary nature, an important point to remember), how do these micro-conscious^2^ entities combine into the higher-level and obvious consciousness we witness in entities like humans and other mammals?

This set of questions is known as the “combination problem,” another now-classic problem in the philosophy of mind, and is what we describe here as the “easy part” of the Hard Problem (Chalmers, 2017). Our characterization of this part of the problem as “easy” is, of course, more than a little tongue in cheek. The authors have discussed frequently with each other what part of the Hard Problem should be labeled the easier part and which the harder part. Regardless of the labels we choose, however, this paper focuses on our suggested solution to the combination problem.

Various solutions to the combination problem have been proposed but none have gained widespread acceptance. This paper further elaborates a proposed solution to the combination problem that we first described in Hunt (2011) and Schooler et al. (2011). The proposed solution rests on the idea of resonance, a shared vibratory frequency, which can also be called synchrony or field coherence. We will generally use resonance and “sync,” short for synchrony, interchangeably in this paper. We describe the approach as a general resonance theory of consciousness or “general resonance theory” (GRT). GRT is a field theory of consciousness wherein the various specific fields associated with matter and energy are the seat of conscious awareness at various levels of organization.

A summary of our approach appears in Supplementary Appendix 1. A summary of our axioms and conjectures appears in Table 1.

**TABLE 1:** Axioms and conjectures of General Resonance Theory.

Axiom 1: All physical entities resonate (“the resonance axiom”)
Axiom 2: All physical entities have some accompanying subjectivity/consciousness (“the panpsychism axiom”)
Axiom 3: Resonating structures in proximity to each other will achieve a shared resonance if the coupling constant is reached or exceeded (“the coupling axiom”)
Conjecture 1: Shared resonance is what leads to the combination of micro-conscious entities into macro-conscious entities (“the shared resonance conjecture”)
Conjecture 2: The boundaries of a macro-conscious entity depend on the velocity and frequency of the resonance chains connecting its constituents (“the boundary conjecture”)
Conjecture 3: Any biological macro-conscious entity will have various levels of subsidiary/nested micro- and macro-conscious entities (“the nested consciousness conjecture”)

### All Things Resonate in Some Manner

All things in our universe are constantly in motion, in process. Even objects that appear to be stationary are in fact vibrating, oscillating, resonating, at specific frequencies.^3^ So all *things* are actually *processes*. Resonance is a specific type of motion, characterized by synchronized oscillation between two states.

**FIGURE 1:**
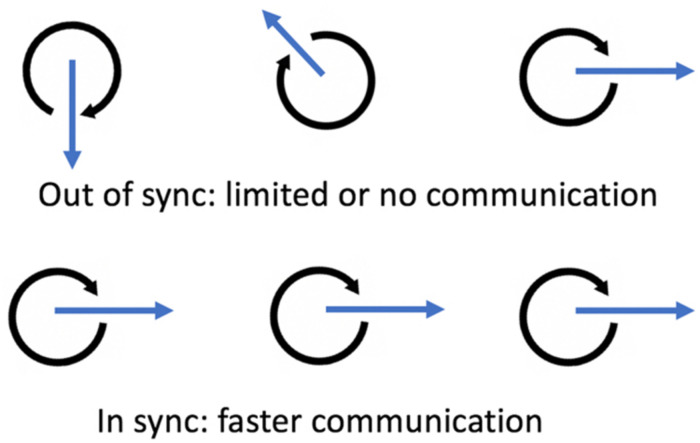
In any set of oscillating structures, such as neurons, shared resonance (sync) leads to increased and faster energy/information flows (the blue arrows) because energy/information flows work together, in “sync,” and are thus amplified (coherent) rather than being “out of sync” (incoherent). Fries (2015) states as an example: “In the absence of coherence, inputs arrive at random phases of the excitability cycle and will have a lower effective connectivity.” The figure offers a schematic view of three oscillators out of sync and in sync.

An interesting phenomenon occurs when different vibrating processes come into proximity: they will often start vibrating together at the same frequency (Strogatz, 2003). They “sync up,” sometimes in ways that can seem mysterious, and allow for richer and faster information and energy flows (Figure 1 offers a schematic). Examining this phenomenon leads to potentially deep insights about the nature of consciousness in both the human/mammalian context but also at a deeper ontological level.

Strogatz (2003) provides various examples of resonance from physics, biology, chemistry, and neuroscience to illustrate “sync” (synchrony), including:

• Fireflies of certain species start flashing their bioluminescent fires in sync in large gatherings of fireflies.
• Large-scale neuron firing can occur in human brains at specific frequencies, with mammalian consciousness thought to be commonly associated with various kinds of neuronal synchrony (Crick and Koch, 1990; Koch, 2004; Fries, 2005, 2015; Dehaene, 2014).
• Lasers are produced when photons of the same power and frequency are emitted together.
• The moon’s rotation is exactly synced with its orbit around the Earth such that we always see the same face.

Let’s delve a little deeper into the idea of resonance.

## What is Resonance, What is Sync?

What’s happening in these examples just provided? Again, resonance/sync is a tendency for different processes to move together – to oscillate – at the same or similar frequency. The science of sync – sometimes called complex network theory or harmonic oscillator theory – is concerned with how coupled oscillators behave in relation to each other. We discuss these theoretical approaches further below.

The question in many examples of resonance is twofold: (1) how do the constituents of each resonating structure (a term we’ll use to refer to any collection of resonating constituents) communicate with each other, and; (2) how do these constituents achieve resonance once that communication occurs?

Let’s look at each question in turn, focusing mostly on the first two examples we listed above: (1) Fireflies synchronizing their flashes; and (2) Large-scale neuronal synchrony in mammalian brains.

### How Do Resonating Structures Communicate?

The nature of communication between each resonating structure will depend on what example we’re considering, and in many cases of resonance the state of the science is still too nascent to offer definitive answers. Looking at fireflies as one of two illustrative examples of complex resonating structures, we know that visual cues are available to each fly but there are probably also olfactory and other chemical cues available, and maybe even electrical or magnetic clues. This kind of sync will require empirical investigation to rule out candidates for communication in order to home in on the channels that are in fact being used by fireflies. From empirical research to date it seems likely that sync in firefly populations that coordinate their bioluminescent flashing relies mostly on visual perception (Strogatz, 2003).

The nature of the communication between each neuron in the case of neuronal synchrony in brains is less clear. Walter Freeman has argued, based on his extensive work on rabbit and cat brains, that electrical field gamma synchrony, a particular type of neuronal sync, is achieved too quickly to depend only on electrochemical neuronal signaling, and must thus also depend on electrical field signaling. Freeman and Vitiello (2006) states:

High temporal resolution of EEG signals … gives evidence for diverse intermittent spatial patterns … of carrier waves that repeatedly re-synchronize in the beta and gamma ranges in very short time lags over very long distances. The dominant mechanism for neural interactions by axodendritic synaptic transmission should impose distance-dependent delays on the EEG oscillations owing to finite propagation velocities and sequential synaptic delays. It does not.

Hameroff (2010) mirrors this conclusion: “The seemingly instantaneous depolarization of gap-junction-linked excitable membranes (i.e., despite the relative slowness of dendritic potential waves or spikelets) suggests that even gap junction coupling cannot fully account for the precise coherence of global brain gamma synchrony.”

Additional research is necessary to further examine the communication channels responsible for achieving gamma and beta synchrony (a less rapid frequency than gamma synchrony), but, as will be discussed, data already available strongly suggest that shared resonance is key for human and other mammalian consciousness. We discuss further below the various types of resonance patterns in mammalian brains, including electrical field resonance, and other recent developments in this scientific field.

### How Do Resonating Structures Achieve Shared Resonance?

We have introduced the first question concerning resonance, without offering any broad solutions at this point. Our second question may be even more complex: How do resonating entities that are in mutual communication adjust their resonance frequencies to achieve resonance with each other? Entities start out of sync and somehow, in many cases, become synced. What forces are at work in these processes?

With respect to fireflies syncing up their flashes, and the mechanisms that allow this kind of sync to occur, we may analogize to human conscious actions. For example, when we want to lift our finger we achieve this intended result through a chain of neural pulses from our brain to our finger, and the motion is achieved. Similarly, it is plausible to speculate that fireflies intend to flash their lights and that after they do so an electrochemical pulse travels from the fly brain to its abdomen. Then the physiological and chemical processes responsible for the fly’s bioluminescence result.^4^

It may seem strange to some readers to ascribe intention to fireflies. To us, however, it seems intuitive and logical that fireflies would experience intentions and conscious control of at least some of their bodily processes – particularly significant processes involving large organs like their light-making organ. Their behavior is complex and displays many “behavioral correlates of consciousness.” We don’t need to suggest, however, that fireflies have anything like the richness of human consciousness to acknowledge, by examining the various neuronal and behavioral analogies to humans and other mammals, that the firefly probably enjoys a rather basic level of conscious awareness.

Under this assumption – that fireflies enjoy a rudimentary type of consciousness, at least in comparison to human consciousness – we may explain the question (how are flashes synchronized?), at a high level, ignoring the complexities of the sub-level mechanisms. This allows for a parsimonious explanation in keeping with how we would explain any conscious action in a human, dog, cat, etc.: the brain/mind wills it and the body responds.

We can also, however, explain firefly flashing sync without recourse to consciousness or intelligence. This is Strogatz’s approach and he and his colleagues first explained the then-mystery of firefly sync by positing internal biological oscillators that automatically sync with neighbors (Strogatz, 2003).

But what about resonance between individual neurons, looking at our second example of synchrony? It would be hard to make the case that individual neurons “intend” to sync up, though there is certainly a case for neuronal-level consciousness of some sort, however, rudimentary it may be compared to whole-brain consciousness. So how do neurons sync up so quickly and so frequently? This remains a mystery but there are various clues that suggest field effects of various types. We don’t know yet how this communication is manifested in each neuron in a way that rapidly changes the electrical cycles in each neuron to match rapidly changing macroscopic patterns.

Keppler (2013) focuses on the phase transitions observed in mammalian brains and the fact that these brains seem to exist generally in a state of “criticality,” which makes them very sensitive to small changes:

From this perspective, the brain mechanisms behind conscious processes can be regarded as a complex system that operates near a critical point of a phase transition. While displaying spontaneous activity and irregular dynamics in the disordered phase, an appropriate stimulus can transfer the brain to the ordered phase that exhibits long-range correlations and stable attractors.

The concept of “phase transition” is potentially quite important in the context of macro-consciousness and the combination problem that is the focus of the present paper. A good analogy to a phase transition is water turning into ice, or water vapor condensing into drops as mentioned above. Small changes in temperature can tip cold water into forming ice crystals that rapidly spread. Similarly, these authors are suggesting that brain states (and the neural states that comprise brain states) can oscillate back and forth quickly, based on relatively modest stimuli. Dehaene (2014) mirrors the notion that phase transitions are highly important in mammalian consciousness. We discuss this further below.

Fries (2015), a major update to Fries’ now well-known “communication through coherence” hypothesis described Fries (2005), frames a cognitive frequency triad of specific electrical brain wave combinations as follows:

The experimental evidence presented and the considerations discussed so far suggest that top–down attentional influences are mediated by beta-band synchronization, that the selective communication of the attended stimulus is implemented by gamma-band synchronization, and that gamma is rhythmically reset by a 4 Hz theta rhythm.

The brief overview of resonance in nature just provided is meant to introduce a number of key ideas that we’ll flesh out below: (1) all aspects of nature are processes rather than static things; (2) all processes/things resonate at various frequencies; (3) processes that resonate in proximity to each other will in some cases sync up and resonate together after a certain time.

These are all components in our approach to resolving the “easy part” of the Hard Problem, otherwise known as the combination problem, which is the focus of this paper. Before we flesh out this solution further, however, we will briefly focus on the “hard part” of the Hard Problem in the next section.

Table 1, presents the axioms and conjectures of our theory, discussed further below.

## The “Hard Part” of the Hard Problem

Chalmers (1996) described what he thought would be required of the eventual “psychophysical laws” governing the relationship between mind and matter – which would collectively comprise the ultimate solution to the “hard problem” of consciousness:

[T]he cornerstone of a theory of consciousness will be a set of psychophysical laws governing the relationship between consciousness and physical systems. … [A]n account of these laws will tell us just how consciousness depends on physical processes. Given the physical facts about a system, such laws will enable us to infer what sort of conscious experience will be associated with the system, if any.^5^

Chalmers’ suggested psychophysical laws would constitute a solution to what he described as the “hard problem” of consciousness (now generally capitalized), which is a new name for the classic mind/body problem.

Hunt (2011) proposed a set of psychophysical laws that would describe the relationship between consciousness and physical systems. The present paper is a follow-up to the earlier work, also discussed in Schooler et al. (2011), and an elaboration of the manner in which resonance plays a key role in achieving macro-scale consciousness through the combination of many micro-conscious entities at various levels of organization.

The first step in any resolution of the Hard Problem requires taking a position with respect to the interaction of mind and matter. As mentioned, our preferred approach, accepts that all matter has some associated mind. This position is often described as panpsychism or panexperientialism (Hunt, 2011, 2014; Schooler et al., 2011; Schooler, 2015; Goff, 2017). In the vast majority of matter this associated mind is very rudimentary – perhaps just a rudimentary humming of simple awareness in, for example, an electron or an atom. But in some types of collections of matter, such as the complex biological life forms we are intimately familiar with, consciousness appears to become dramatically more rich in comparison to the vast majority of matter (Koch, 2014; Tononi and Koch, 2015).

Based on the observed behavior of the entities that surround us, from electrons to atoms to molecules to bacteria to paramecia to mice, bats, rats, etc., all things should be viewed as at least a little conscious. Panpsychism represents the counterpoint to the *emergentist* viewpoint, which argues that consciousness emerged at a particular point in the development of each species that enjoys consciousness, and also emerged at a particular point in the development of each organism that enjoys consciousness, where it wasn’t before.

The panpsychist argues, rather, that mind did not emerge; it’s always associated with matter and *vice versa* (they are two coequal sides of the same coin), but the mind that is associated with all matter is generally extremely rudimentary. An electron or an atom enjoy just a tiny amount of consciousness. But as matter complexifies, so mind complexifies, and *vice versa*. It is not, however, any kind of increase in complexity that matters in this context. It is, rather, a function of greater resonant interconnections, both internally and externally. Hunt (2019) fleshes out the mathematical framework first described in Hunt (2011), focusing on how resonant connections lead to larger-scale conscious entities and how such entities may be characterized and quantified.

We won’t delve too deeply into the many arguments in favor of panpsychism here, but *see* Griffin (1998), Hunt (2011, 2014), Schooler et al. (2011), Schooler (2015), and Goff (2017). Christof Koch, a pioneer in the scientific investigation of the neural basis of consciousness, wrote a short piece in 2014 explaining his “coming out” as a panpsychist (Koch, 2014):

Elementary particles either have some charge, or they have none. Thus, an electron has one negative charge, a proton has one positive charge and a photon, the carrier of light, has zero charge. As far as chemistry and biology are concerned, charge is an intrinsic property of these particles. Electrical charge does not emerge from non-charged matter [it just is there]. It is the same, goes the logic, with consciousness. Consciousness comes with organized chunks of matter. It is immanent in the organization of the system. It is a property of complex entities and cannot be further reduced to the action of more elementary properties. We have reached the ground floor of reductionism.

Koch has also partnered with Giulio Tononi in developing the Integrated Information Theory of consciousness, which is panpsychist in its assumptions (Tononi, 2012; Oizumi et al., 2014; Tononi and Koch, 2015).

Our preferred stance is panpsychist because we recognize the major difficulties with emergentist materialism (the prevailing view among philosophers and physicists; though the tide is turning toward panpsychism) and it seems more plausible that, just as life evolves smoothly from one form to another, so mind evolves smoothly from one form to another – in fact these processes are concurrent and inter-related.

We won’t dwell on the debate about various metaphysical foundations for explaining consciousness, however, in this paper. Rather, we take panpsychism as our starting point and we will focus on the “easy part” of the Hard Problem – the combination problem, described further in the next section.

Summing up: arriving at some version of panpsychism constitutes a solution to the “hard part” of the Hard Problem that we find more convincing than the alternatives.

## The “Easy Part” of the Hard Problem

The “easy part” of the Hard Problem is, as discussed above, more generally known as the “combination problem” or the “binding problem” (Chalmers, 2017). The combination problem refers to the question of how different micro-entities combine to form a higher-level macro-conscious entity. That is, how do the purported experiences in, say, individual neurons, or regions of the brain, combine together to create a larger-scale experience that is still individual and unitary?

Goff (2017) states the problem well: “We feel we have some kind of grip on how. parts of a car engine make up an engine, but we are at a loss trying to make sense of lots of ‘little’ (proto) minds forming a big mind.”

This “combination problem” is not unique to panpsychism, as is often suggested. It is a problem in every reductionistic answer to the Hard Problem, whether we are materialist (see, e.g., Dehaene, 2014, detailing one researcher’s many years trying to determine how parts of the brain combine to form consciousness in an explicitly materialist framework) or panpsychist in our approach. This is the case because any reductionist explanation of consciousness must explain how the components of a given brain or mind (or brain/mind) combine to form a seemingly unitary consciousness. None of these approaches has achieved any consensus with respect to this basic problem.

William James first described what is now known generally as the combination problem or the boundary problem in his 1890 book *The Principles of Psychology* (James, 1890):

Where the elemental units [of our theory of mind] are supposed to be feelings, the case is in no wise altered. Take a hundred of them, shuffle them and pack them as close together as you can (whatever that may mean); still each remains the same feeling it always was, shut in its own skin, windowless, ignorant of what the other feelings are and mean. There would be a hundred-and-first feeling there, if, when a group or series of such feelings were set up, a consciousness belonging to the group as such should emerge. And this 101st feeling would be a totally new fact; the 100 original feelings might, by a curious physical law, be a signal for its creation, when they came together; but they would have no substantial identity with it, nor it with them, and one could never deduce the one from the others, or (in any intelligible sense) say that they evolved it.

Our resonance theory and empirical data suggest, however, that these “elemental units” in James’ passage aren’t “windowless.” Rather, all processes are constantly interacting with other processes nearby; they do in fact have “windows” that allow for such interaction, and when full resonance occurs these windows are maximally open (see Figure 1 for a schematic depiction).

We suggest, based on the growing body of data described sketchily above, that a shared resonance frequency is the key to resolving the combination problem, the “easy part” of the Hard Problem. Resonating entities bind/combine together in various ways when they resonate at the same frequency. Depending on the entities being considered, such shared resonance and binding can in certain circumstances lead to a combination of mental qualities into a larger unified whole. We address the various versions of the combination problem (Chalmers, 2017) in Supplementary Appendix 2.

What is it about shared resonance that would allow such a combination or binding of mental qualities? It is clear today that the Democritean/Newtonian notion of matter as akin to tiny billiard balls careening around in space is highly incomplete as a model of reality. We know from quantum field theory and abundant data over the last century that matter is at its fundamental level more like a very small standing wave of concentrated energy. Each of these waves are more or less localized in space and time.

When we see matter as fundamentally wave-like, it is not hard to see why shared resonance leads to faster energy and information flows, and resulting macro-conscious entities. All physical processes are, at least in part, different types of waves, so when various entities resonate at the same frequency the waves work together, instead of in opposition. This allows for significantly higher bandwidth and speed in the energy and information flows between the constituents of whatever resonating structure we’re looking at. The resonating wave forms of the micro-conscious entities are then coherent and information flows combine into a larger entity, a larger harmonic, rather than occurring out of phase (decoherently).

Biophysicist Christof Koch hinted at this approach in his 2004 book, *The Quest for Consciousness*:

If the input [to the brain] is more sustained and is boosted by top-down attention … a sort of standing wave or resonance might be created in the network, with vital contributions from the feedback pathways. Both local and more global feedback could cause neurons to synchronize their spiking activity above and beyond the degree of synchronization that results from the sensory input by itself. This increases their postsynaptic punch compared to when they fire independently. A powerful coalition of neurons could be assembled in this manner, able to project its influence to the far reaches of the cortex and below. This would be the slow mode that underlies conscious perception.

Fries (2005, 2015) follow on from Koch’s suggestions by fleshing out a “communication through coherence” hypothesis. Fries (2015) provides a helpful example of shared resonance in the context of neuronal resonance/coherence: “In the absence of coherence, inputs arrive at random phases of the excitability cycle and will have a lower effective connectivity.” Conversely, inputs that arrive synced to the same excitability cycle will propagate faster and with greater bandwidth. We look further at Fries’ model below.

**FIGURE 2:**
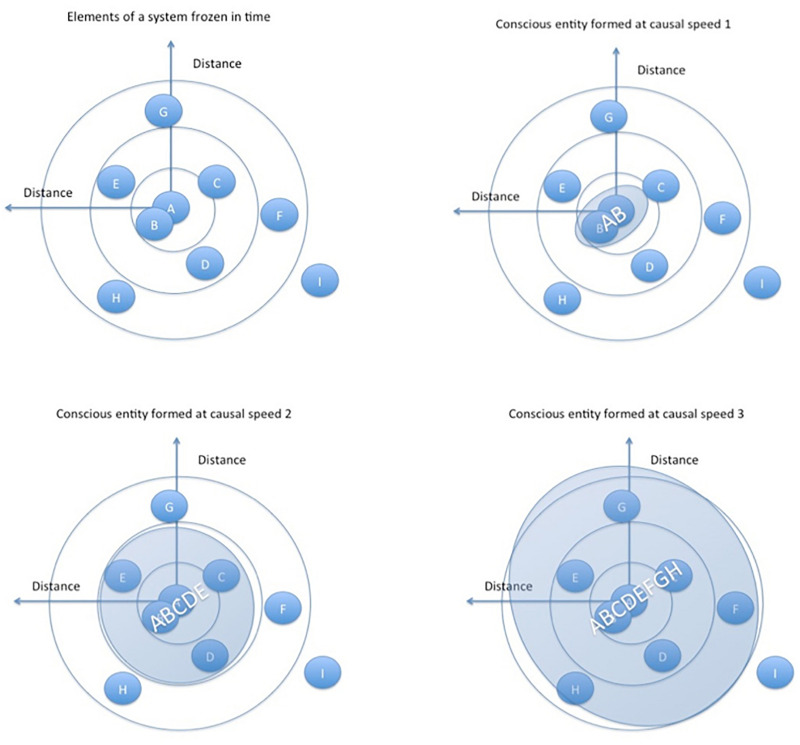
Based on GRT, the speed of causal (energy/information) flows leads to larger and more complex conscious entities through shared resonance (this is our Conjecture 2, discussed further below). Shared resonance allows the constituents to “sync up” into a coherent whole, achieving a phase transition in energy/information flows. Speeds 1, 2, and 3 are different speeds of causal/energy/information flows between the abstract entities, which lead to different constituents forming the larger resonating whole in each example. Larger resonating entities form as a result of higher energy/information speeds. The combined entity AB is formed at causal speed 1 in the top right image, and at causal speed three in the lower right entity ABCDEFGH is formed.

Biological organisms have leveraged faster information exchange through various biophysical information pathways. These faster and richer information flows allow for more macro-scale levels of consciousness to occur than would occur in similar-scale structures like boulders or a pile of sand, simply because there is greater interconnectivity and thus more “going on” in biological structures than in a boulder or a pile of sand (Figure 2). However, as stated previously, the type of interconnectivity must be based on resonance mechanisms that, as a general matter, induce a phase transition in the speed of information flows due to the transition from incoherent structures to coherent structures. Boulders and piles of sand amount to “mere aggregates” or just collections of highly rudimentary conscious entities (perhaps at the atomic or molecular level only), rather than combinations of micro-conscious entities that combine into a higher level macro-conscious entity, as is the case with biological life.

The *type* of interconnection and combination between resonating structures is key for consciousness to expand beyond the highly rudimentary type of consciousness that we expect to occur in an electron, atom, or molecule. The central thesis of our approach is this*: Shared resonance among micro-conscious constituents allows for macro-conscious entities to arise because of a phase transition in the speed and bandwidth of information exchange*. We flesh out this idea, what we refer to as Conjecture 1, in the rest of the paper.

In human brains, for example, we’ve seen already that one candidate for the primary neural correlate of consciousness is some type of gamma synchrony (Dehaene, 2014, focused on various “signatures of consciousness,” including late-onset gamma synchrony; Hameroff, 2010), but mammalian consciousness is generally correlated with a combination of lower harmonic frequencies as well as gamma (Fries, 2015).

This shared resonance through specific neuronal electrochemical firing patterns creates an electromagnetic field that may itself be the seat of macro-conscious awareness (*see also* for electromagnetic field theories of consciousness Pockett, 2000; John, 2001; McFadden, 2002a, b, 2013; Jones, 2013). Looking only at gamma synchrony, as an example for discussion: as the area of shared gamma synchrony (what Hameroff, 2010 calls “the conscious pilot”) moves around the brain, supported by other slower frequency waves (Fries, 2015), it absorbs new neurons into the same resonance frequency; and as it moves away from certain neurons it allows them return to their previous state of resonance corresponding to a more localized pattern.

By absorbing new neurons into the moving semi-stable gamma wave pattern, this moving large-scale wave entrains those neurons to the same frequency and thus allows information and processing power of the many micro-conscious entities constituted by those neuron clusters to become part of the macro-conscious entity, and also to achieve a phase transition in the speed of information exchange by moving from primarily electrochemical information exchanges to electromagnetic field exchanges, which are significantly faster. Through this entrainment, the smaller-scale harmonics are entrained into the larger harmonic – the constituents’ “windows” are all open to each other and information flows more freely (see Figure 1 for a schematic). The macro-consciousness is changing in each moment to exactly the degree that its constituent neurons, and associated fields, are changing in each moment.

Whitehead, one of the most influential panpsychists of the modern era, states in his 1929 magnum opus, *Process and Reality*, that “the many become one and are increased by one” (Whitehead et al., 1929, p. 21). This means that a new higher-level entity arises from the lower level of entities, but the lower-level entities are not eliminated in this binding process; there is binding of the lower-level entities into a new entity, and thus an increase of one. The many become one and are increased by one. As the “conscious pilot” moves around the brain it includes various subsidiary micro-conscious entities at various levels of organization, combining them into a single dominant consciousness without extinguishing the subsidiary consciousnesses.

Table 2 shows various information pathways in mammal brain, with their velocities, frequencies, and distances traveled in each cycle, which is calculated by dividing the velocity by the frequency. These are some of the pathways available for energy and information exchange in mammal brain and will be the limiting factors for the size of any particular combination of consciousness in each moment.

**TABLE 2:** Various energy pathway velocities and frequencies in mammal brains.

**Energy pathway**	**Velocity**	**Frequency**	**Distance traveled per cycle**
Terahertz-level tubulin resonance (Craddock et al., 2017)	Possibly > c	613 THz	0.0049 m
Electrochemical pulses through axons (Siegel et al., 2019)	80–120 m/s	Various	Various
Gamma waves in human whole brain	10 m/s	∼40 Hz	0.25 m
Theta waves in human whole brain (Zhang and Jacobs, 2015)	3 m/s	∼5 Hz	0.6 m
Beta waves in human whole brain (Takahashi et al., 2011)	0.23 m/s	∼25 Hz	0.0092 m
Weak electric fields (“ephaptic coupling”) (Chiang et al., 2019)	0.1 m/s	<1 Hz	∼0.1 m
Gap junction sharp wave “ripples” in mouse brain (Maier et al., 2003)	0.016 m/s	200 Hz	0.00008 m

## “Consciousness Through Shared Resonance” Compared to Fries’ Communication Through Coherence Model

Our consciousness-through-shared-resonance proposal has some recent precedents. Fries first proposed his Communication Through Coherence (CTC) model in 2005 and issued a substantial update to his theory in 2015 (Fries, 2015). The update summarized the relevant research in the intervening decade, and also modified the original theory in light of contrary evidence. Our approach mirrors in some ways Fries’s CTC model, and other similar approaches, in terms of a proposed model for human cognition and consciousness.

Our aim, however, in this paper and our previous work is to generalize insights from the study of mammalian consciousness to a broader theory about consciousness and ontology that applies beyond mammalian consciousness, potentially to all physical entities (this is what panpsychism means in this context).

Fries (2015) describes his CTC model and the role of “selective communication” as a function of resonance between different components of the brain (what we described above as the “windows” that are opened when coherent/resonant, and closed when incoherent/not resonant):

[S]trong effective connectivity requires rhythmic synchronization within pre- and postsynaptic groups and coherence between them, or in short—communication requires coherence. In the absence of coherence, inputs arrive at random phases of the excitability cycle and will have a lower effective connectivity. A postsynaptic neuronal group receiving inputs from several different presynaptic groups responds primarily to the presynaptic group to which it is coherent. Thereby, selective communication is implemented through selective coherence.

Coherence also allows for entrainment of certain neurons to the dominant resonance frequency (*id.*):

In addition to rendering communication effective and precise, coherence also renders communication selective. If one set of synaptic inputs, constituting one neuronal representation, succeeds in triggering postsynaptic excitation followed by inhibition, this inhibition closes the door in front of other inputs. Those other inputs are then unable to transmit the neuronal representation that they constitute, and they are unable to trigger inhibition themselves. Thereby, the winning set of synaptic inputs conquers the perisomatic inhibition in the postsynaptic neuronal group, entrains it to its own rhythm, and thereby establishes a communication link that is selective or in other words, exclusive.

Demertzi et al. (2019) generally reflects this view in highlighting the role of evolving dynamic processes in consciousness: “the neural correlates of consciousness could be found in temporally evolving dynamic processes, as postulated by influential theoretical accounts.” Dehaene (2014) cites Fries (2015) frequently with respect to the notion that synchrony/coherence have a strong role in the dynamics of consciousness in humans.

Zeki and Bartels have also suggested an approach that shares certain features with the model proposed here. It is now widely accepted that evolution entailed a process in which simple organisms combined to form the organelles (e.g., mitochondria) of more complex eukaryotic cells, which in turn combined to become multi-cellular organisms (Margulis and Sagan, 1990). Just as life evolved the capacity to integrate independent living creatures into more complex singular life forms, it may have similarly developed the capacity to integrate subjective experiences into nested hierarchies of higher-order conscious entities. Such a hierarchical view of consciousness represents the basis of Zeki and Bartel’s (Zeki and Bartels, 1999; Zeki, 2003) theory of how consciousness manifests in the brain. We share this view of nested hierarchies representing the structure of consciousness, with the difference that we posit far more than the three levels of consciousness that Zeki posits.

Drawing on differences in the processing rates of different areas of the visual system, Zeki suggests that the brain engages in a nested hierarchy of distinct conscious experiences leading to a final unified experience. He proposes three hierarchical levels at which consciousness takes place in the brain: micro-consciousness corresponding to the different levels of the visual system that process distinct attributes (e.g., V4 processes color where as V5 processes motion), macro-consciousness that integrates multiple attributes of a system (e.g., binding color to motion), and unified consciousness corresponding to the experience of the perceiving person. Zeki further suggests that each of these nested levels of consciousness occur in a distinct temporal order, with the lower order levels being ahead of and feeding into the higher order levels. Zeki describes his model as follows (Zeki, 2003):

It thus becomes possible to distinguish three hierarchical levels of consciousness: the levels of micro-consciousness, of macro-consciousness, and of the unified consciousness. Of necessity, one level depends upon the presence of the previous one. Within each level, one can postulate a temporal hierarchy. This has been demonstrated for the level of microconsciousness, because color and motion are perceived at different times. It has also been demonstrated for the level of the macro-consciousnesses, because binding between attributes takes longer than binding within attributes… Micro- and macro-consciousnesses, with their individual temporal hierarchies, lead to the final, unified consciousness, that of myself as the perceiving person.

Although Zeki only describes three levels, we suggest here that there are likely many additional lower-level micro-conscious entities that are part of the human/mammalian hierarchy of consciousness.

Accordingly, the inorganic world may involve only the most micro-level conscious observers at the level of atoms and molecules. In contrast, life may have evolved the capacity to develop hierarchies of conscious observers within observers, based on the foundational level of (highly rudimentary) consciousness present within atoms and molecules, with each level subsuming a more macroscopic perspective. This process leads ultimately to the highest level at which the unified dominant experience of the organism occurs. This nested hierarchy is likely far larger than just three levels of hierarchy, but Zeki’s approach makes sense as a schematic.

Zeki offers a way of distinguishing his proposed levels, namely, by the temporal order in which they occur, with higher order experiences occurring temporally downstream. In other words, Zeki’s view suggests that the different conscious observers in the brain may experience the same events at slightly different times and at different durations, with the final unified consciousness entailing the longest moments/durations of experience.

## What Type of Resonance is Necessary for Combination of Consciousness?

We have already suggested the mechanism for combination to occur: a shared resonance leads to combination and as a result the “many become one and are increased by one.” This means that each micro-conscious entity is, upon achieving a shared resonance, included in a new higher-level conscious entity, but the micro-conscious entities are not extinguished through such combination. The new macro-conscious entity supervenes on the micro-conscious entities.

But what type of resonance leads to such combination? What energy/information pathways must achieve a shared resonance for the combination of consciousness to occur. Confining our consideration for now to mammalian consciousness, we have good data to support the suggestion that it is a shared electrical resonance (at various frequencies) that is generally the proximate cause of combination, as discussed above (Fries, 2005, 2015). There may also be a shared quantum entanglement resonance that *precedes and leads to this shared electrical resonance*, or that has some other relationship to the shared electrical resonance. We turn this to issue in the next section.

We also note that physical structures can remain largely the same while acting as the substrate for vastly different states of consciousness, due to the ever-changing flows of energy/information that supervene on the generally stable physical structures. Fries (2015) states this proposition well – referring to stable structures as the “backbone” of conscious processes: “If neuronal communication depends on neuronal synchronization, then dynamic changes in synchronization can flexibly alter the pattern of communication. Such flexible changes in the brain’s communication structure, on the backbone of the more rigid anatomical structure, are at the heart of cognition.”

In other words, physical structures can fail to produce macro-consciousness if the shared resonance states remain highly localized. Our Figure 1 shows schematically why this is the case: disordered, non-resonant states simply don’t allow for the sharing of information across much distance. It is large-scale shared resonance that is key to the dominant consciousness that humans enjoy as normal waking consciousness, for example. Critically, however, there is a hierarchy of resonances in neural systems (Steinke and Galán, 2011; Riddle and Schooler, 2019) such that lower-level resonance (entailing less information integration) may still take place even while higher-level resonances (corresponding to greater degrees of integration) are absent, either temporarily (sleep, seizure, etc.) or permanently (death, in which case lower-level resonances may continue for some time but will also, before too long, dissipate).

These dynamics may explain why during seizures large areas of the brain can be synchronized at local and regional scales without conscious experience at the global level. Accordingly, during absence seizures lower-level system (individual neurons and local clusters of neurons) are in synchronization but higher levels of organization lose their distinct synchronization and thus reportable conscious states are generally not possible (though there is considerable debate in this area with respect to absence seizures and regional synchronization: Jiruska et al., 2012).

Our approach also explains why substantial changes in consciousness occurs so regularly without substantial changes to Fries’ “backbone.” For example, in NREM-3 sleep, unresponsive wakefulness states, absence seizures, or general anesthesia, the anatomical structure isn’t changed significantly; yet the states of consciousness are dramatically different. Our resonance theory suggests that this is because the energy/information flows, made possible by various types of shared resonance, have also changed dramatically. Resonance reflects the dynamics of the physical structures, so the generally stable physical structure can remain stable while the states of resonance, and thus states of consciousness, can and do change substantially.

### The “Quantum Question”

Some comments on the controversy about claims of quantum phenomena in the biological context are important before we delve into details of quantum biology. There has been a long debate about whether quantum phenomena are even possible in biological systems (Tegmark, 2000; Hameroff and Penrose, 2014; Craddock et al., 2017). Some physicists and neuroscientists adopt the view that quantum phenomena cannot be present in the warm wet systems of mammalian brains. However, the field of “quantum biology” is thriving and new examples of established or potential quantum effects in warm wet biological systems are coming to light with increasing regularity (see Lambert et al., 2013, for a review). In sum: it is now well-established, despite the commonly held view that quantum effects can’t be present in mammalian brains, that quantum phenomena are in fact quite common in biological systems.

While we are open to the possibility of quantum phenomena in mammalian brains, and possibly being part of the complex causal phenomena of consciousness, our resonance theory of consciousness does not require the presence of quantum phenomena for its validity. Our concern in raising the “quantum question” is twofold: (1) to not rule out quantum phenomena prematurely (to follow the facts where they may lead), but also (2) to not rush to the conclusion that quantum phenomena are in fact present in the dynamics of consciousness. There is plenty of room between accepting, for example, that some brain dynamics seem to operate independently of traditional electrochemical neuronal pathways, and the notion that quantum phenomena must be invoked to explain such apparent anomalies. There is a vast middle ground that should be explored before such an alternative explanation is considered to be necessary.

What is happening that seems to allow such rapid communication across large parts of mammalian brains? Freeman and others (Pockett, 2000; McFadden, 2001, 2002a,b, 2013) have suggested that the electric field itself, which is created by the brain and supervenes on the brain, becomes a mediator of information (for recent and interesting data about slow oscillating electric field coupling allowing non-synaptic neural communication, see Chiang et al., 2019), and that cortical neurons that resonate together undergo a phase transition into a unitary quantum state. Freeman and Vitiello (2006) states:

Our field theoretic model leads to our view of the phase transition as a condensation that is comparable to the formation of fog and rain drops from water vapor, and that might serve to model both the gamma and beta phase transitions.… The adoption of such a field theoretic approach enables us to model the whole cerebral hemisphere and its hierarchy of components down to the atomic level as a fully integrated macroscopic quantum system, namely as a macroscopic system which is a quantum system not in the trivial sense that it is made, like all existing matter, by quantum components such as atoms and molecules, but, as already mentioned, in the sense that some of its macroscopic properties can best be described with recourse to quantum dynamics.

Weingarten et al. (2016) shows plausible pathways for quantum networks to exist alongside classical networks in the brain.

The current evidence suggests that it is plausible that electrical resonance is mediated by quantum resonance and perhaps prompted/prodded by quantum resonance in such a way that the very rapid achievement of electrical resonance states that we observe in mammalian brains is made possible by the far more rapid quantum resonance states at the subcellular level.

We are agnostic currently about the likelihood or necessity for quantum mechanisms mediating mammalian consciousness, or for the necessity of quantum mechanisms to be involved in a broader explanation of consciousness beyond the category of mammalian brains. At this juncture there is not strong evidence that an ongoing quantum synchrony is the pathway for shared information between the constituent micro-conscious entities. That appears to primarily occur through electrochemical pathways.

Regardless of whether quantum mechanisms are required to understand mammalian consciousness, however, the theory and worldview we are proposing here is consonant with the quantum mechanical worldview and its wave-and-field-based ontology, which is the underlying ontology of today’s physics.

In this ontology, all actual entities exist in a large interconnected web. To exist in this web, or “field” (or set of fields), to use more appropriate physics terminology, is to send waves of various frequencies to the rest of the field. These frequencies are simply waves of the field itself, not something existing over and above the field. The universe consists of only the field and various waves moving through that field. What we think of as matter or energy consist of more concentrated and higher frequency waves of that field.

This is the ontology strongly suggested by quantum field theory, but even though quantum field theory has been with us for half a century, its details are complex and controversial. Accordingly, the field and wave-based ontology that is the core of quantum field theory hasn’t generally sunk into the collective scientific consciousness.

### What Information Pathways Are Responsible for Macro-Scale Consciousness?

We’ve suggested above that electrical and electrochemical forces seem to be the most relevant energy and information pathways for macro-consciousness, with a possible role for quantum interactions. This section looks further into what fundamental forces are relevant to consciousness.

There are various information pathways we should look to in our survey of potential information pathways, starting with the fundamental physical interactions: electromagnetism; gravity; the strong and weak nuclear forces; and the newer but now well-established quantum entanglement interaction.

We suggest the provisional conclusion that characteristics of electricity and electromagnetism more generally seem to make it the most suitable type of resonance for combination beyond the atomic scale, perhaps mediated or enhanced by various types of subcellular quantum resonance.

By definition, the strong and weak nuclear forces generally apply only to nuclear-scale physics, which seems to leave these forces out of the equation in terms of any putative consciousness larger than a nucleus or an atom.

Gravity is the other established long-range force but we have little reason to believe that gravity plays much if any role in consciousness because it is so weak compared to electromagnetism. At the spatial scale of biological organisms that we are familiar with – what we’ll call the “organic scale” from now on – electromagnetic effects vastly overshadow gravitational effects – by many orders of magnitude. Penrose and Hameroff have, however, suggested a role for gravity in ongoing quantum collapse in their recent work revising their Orchestrated Objective Reduction theory of consciousness (Hameroff and Penrose, 2014), based on the proposed Diosi-Penrose objective reduction approach to quantum gravity.

Because electromagnetism is so many orders of magnitude stronger than gravity it seems to be the best candidate currently for the primary resonance pathway at the scale of biological life and macro-consciousness. A growing body of evidence suggests, however, that quantum effects are also operative and influential at the organic scale, as discussed above. The potential complementary relationship between quantum resonance and electrical resonance requires considerably more research, and we can’t say much about how these two forces interact at the organic scale, or whether they are in some sense the same resonance phenomenon with different manifestations – they are perhaps two sides of the same coin.

If we accept the provisional conclusion that electricity or electromagnetism more generally is the place to look for shared resonance between neurons and brain regions, we must still consider the question: what components of neurons and what physical processes in neurons must achieve a shared resonance for the combination of micro-conscious entities to occur?

### Is Subcellular Resonance Key to Consciousness?

A growing body of research examines the subcellular processing taking place in microtubules and other proteins like actin, beta spectrin, SNARE complex and clathrin (Sahu et al., 2013a, b; Craddock et al., 2014, 2015; Hameroff and Penrose, 2014; Bandyopadhyay, 2017). Microtubules and similar proteins are common in neurons and were previously thought to be a type of support scaffolding for these types of cells. Recent research has, however, shown that these molecules can be rich information processors through the activity of dipolar tubulin molecules, at least in the case of microtubules. Hameroff (2013) calculates at least 10^16^ additional operations per second arising from microtubule operations *in each neuron*, which is a massive increase over the roughly 10^5^ operations per second per neuron thought to be possible through axonal-dendritic connections alone.

Hameroff and Penrose (2014) describes an important addition to their Orch OR approach in which “beat frequencies” at classical oscillation time periods manifest during such oscillations, but are based on faster quantum oscillations at a more granular level, thus addressing objections about decoherence occurring at “warm” biological temperatures that some have leveled against Orch OR:

In previous Orch OR publications, the relevant time τ for conscious moments … has been assumed to correlate with physiological EEG parameters, i.e., 10 to several hundred milliseconds, which is relatively long for isolated quantum systems. But here we suggest an alternative way in which such oscillation frequencies might come about, namely as *beat* frequencies, arising when OR is applied to superpositions of quantum states of slightly different energies. This makes the task of finding an origin for these observed frequencies far simpler and more plausible.

Hameroff and Penrose (2014) cite Bandyophadyay’s work on subcellular resonance and name it “Bandyophadyay coherence” as an homage to his work in this area (citing Sahu et al., 2013a, b).

Could a higher frequency shared resonance – well beyond the 30–120 Hz of gamma frequency – of these subcellular processes lead to the combination of consciousness at the subcellular level? Craddock et al. (2017), for example, looks at the correlation between terahertz-level oscillations in tubulin molecules and anesthetics. Or is it only the cellular and inter-cellular levels of resonance that we should look to as the causal mechanisms for combination of consciousness? Or do subcellular processes precede in time the cellular processes, at a more granular scale, which then lead to a shared resonance at the global level of each brain?

These are all questions that should be active areas of research and we are optimistic that research in these areas will grow considerably in the coming years.

### Is There a Resonance “Signature”?

We suggest a corollary to the resonance hypothesis: that each particular mind may enjoy its own resonance signature, a particular frequency that is commonly manifested during waking consciousness. This signature will change slightly in each moment, or will perhaps oscillate around an average frequency value. But, for example, Schooler’s normal gamma synchrony signature is slightly different than his mother’s, or yours, or his cat’s, or a bacterium’s resonance frequency (bacteria don’t experience gamma synchrony as far as we know).

Each particular signature frequency will itself be changing constantly in time, so there is no “essential” signature that constitutes you or us. But as just suggested, there may be average values around which our own individual signatures oscillate.

## Testing the Framework: Does Shared Resonance Necessarily Lead to Combined Consciousness?

In this manuscript, we offer a meta-synthetic framework for conceptualizing how complex conscious experience may emerge in physical systems. Our approach starts by assuming panpsychism as a defensible (and for one of us, Hunt, particularly compelling) approach for addressing the Hard Problem of how consciousness relates to matter.

The version of panpsychism we adopt suggests that all physical entities are accompanied by at least some rudimentary type of consciousness. It is only in more complex physical systems, however, that richer types of consciousness arise. Having adopted this perspective, we argue that the concept of shared resonance could address one of the fundamental challenges (i.e., the combination problem) that has been perceived as a problem for panpsychist approaches.

We suggest below a framework for possible experiments for testing our framework as well as other theories. We are fleshing out the testing/falsification proposals in other work. It is important to note, however, a key methodological and epistemological limitation up-front: all attempts to assess the presence or nature of consciousness in any particular system, and related attempts to assess different theories of consciousness, must rely on reasonable inference rather than the notion of proof or any type of incontrovertibility, because the only consciousness we can know with any certainty is our own. This limitation applies to regular human life as much as it does to the science of consciousness.

This fundamental epistemological problem is surmounted frequently in practice, however, in that we, each of us, reasonably infer that other people are conscious, based on their behaviors, speech, and appearance. The same general approach holds true (for many of us) with respect to pets and various other animals that can’t communicate in words, yet which we still reasonably infer to be conscious based on their behavior.

**FIGURE 3:**
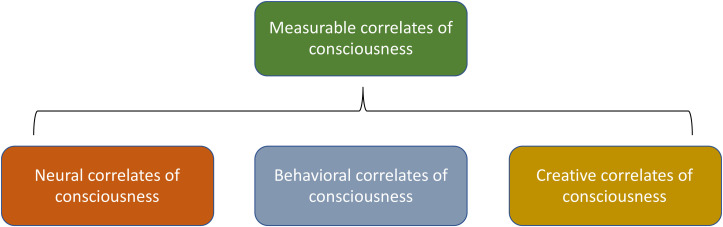
The various types of measurable correlates of consciousness (MCC).

Accordingly, testing consciousness down the chain of biological and physical complexity will rely on making similar reasonable inferences, based on a quantification framework that rests on a general notion of “measurable correlates of consciousness” (MCC, Figure 3). This is a broader notion than “neural correlates of consciousness” (NCC), the physically measurable neural dynamics that are associated with reported conscious awareness in various contexts, or the related but broader notion of “behavioral correlates of consciousness” (BCC), which are the real-time observable behaviors exhibited by candidates for consciousness (Tononi and Koch, 2015).

MCC refers to any reliable means identified for measuring aspects of consciousness. Along with the NCC and BCC, just described, the MCC also includes, in our taxonomy, “creative correlates of consciousness” (CCC), which are the products of consciousness that can be separated spatially and temporally from the conscious entity that created them. The CCC are similar to the real-time behavioral observations that constitute BCC but the CCC may be separated in time and/or space from the creator, whereas thee BCC are by definition real-time and may not be separated in time or space from the creator.

For example, various types of “art,” such as paintings, songs, sculptures, writings, etc., constitute CCC, whether these are human made or not (e.g., does impressive AI-created visual art lead anyone to consider that AI to be conscious? If not now, as AI gets better and better will future similar works, as they increase in complexity and depth, suggest real consciousness in the AI that created them?). The suggestion is that we can learn something meaningful about consciousness by observing the creative products of ostensibly conscious entities, and make judgments about the consciousness (or lack thereof) of an entity capable of creating whatever CCC is being considered.

We must keep in mind that “Finding reliable markers indicating the presence or absence of consciousness represents an outstanding open problem for science.” (Demertzi et al., 2019). Demertzi and colleagues, and various other researchers have been working to identify reliable markers, but this is still a nascent field.

Dehaene (2014) states the problem clearly (p. 211): “[C]ould any brain image ever prove or disprove the existence of a mind?” He answers this question in the affirmative, with various discussions about the neural correlates of consciousness and “signatures of consciousness” (what he considers to be the necessary and sufficient correlates of consciousness), but also recognizes that (p. 214) “no single test will ever prove, once and for all, whether consciousness is present.” He instead recommends a battery of tests be developed to give more confidence about the presence of consciousness in various contexts, focused on human subjects.

Testing the framework presented here should focus initially on the three conjectures in our Table 1. This approach follows the Lakatosian research program (Lakatos, 1968) that focuses on testing the “hard core” principles of any given theory. Conjectures 1–3 in Table 1 are the core of General Resonance Theory. There are many ways that various MCC may be measured to test conjectures 1–3 and we are fleshing out these ideas in other work.

Here again are the three primary conjectures of General Resonance Theory:

Conjecture 1: Shared resonance is what leads to the combination of micro-conscious entities into macro-conscious entities (“the shared resonance conjecture”).

Conjecture 2: The boundaries of a macro-conscious entity depend on the velocity and frequency of the resonance chains connecting its constituents (“the boundary conjecture”).

Conjecture 3: Any biological macro-conscious entity will have various levels of subsidiary/nested micro- and macro-conscious entities (“the nested consciousness conjecture”).

Our conjectures may be tested by using assorted MCCs to discern the information/energy processing characteristics associated with known conscious states and comparing to lower-level systems. It may be especially useful to examine minimally conscious states (induced by drugs or sleep) since these provide a boundary condition for the minimal requirements for consciousness. Accordingly, if shared resonance underpins the combination of consciousness (Conjecture 1), then various resonance chains should be observed in conscious but not unconscious states.

If the boundaries of macro-conscious entities depend on the velocity and frequency of resonant chains (Conjecture 2), this should be reflected in differential access to specific information that is accessible within the cycle time of each information pathway examined.

Finally, if lower-level organizational systems combine into various nested states of consciousness (Conjecture 3), then the synchronization and information integration characteristics between one level to the next at various levels of organization should resemble in key ways that associated with the arising of dominant conscious states in humans and other mammals.

Testing Conjecture 3 will depend on further development of techniques for assessing information/energy flows between levels. Although much work needs to be done in this regard, we are encouraged by the information processing approaches developed in the IIT model (Oizumi et al., 2014) (although see comments below), and related information quantification approaches (Seth et al., 2011; Arsiwalla and Verschure, 2018). We also suspect that other models of information extraction/integration may prove helpful. For example, Fanelli (2019) offers a formal universal model of information compression that might be used to characterize the information extraction associated with minimally conscious states. This compression value might then be used to examine information sharing at lower levels. A prediction of Conjecture 3 is that information compression values should be similar in some ways across organizational levels.

The present model’s consideration of information processing and synchrony in drawing inferences about the nature of consciousness raises comparisons with both Integrated Information Theory (IIT) (Tononi, 2012; Oizumi et al., 2014), and Global Workspace Theory (GWT, Baars, 2005; Dehaene, 2014). We briefly address here some commonalities and differences between General Resonance Theory (GRT), Integrated Information Theory, and Global Workspace Theory.

Hunt (2014) compares IIT to GRT (an earlier version thereof), and suggests adding a “time mechanism” to resolve one of the more serious difficulties with IIT (the exclusion principle and exclusion mechanism), so we will not dwell on this here. The key difference between GRT and IIT, in addition to a process time that is the focus of Hunt (2014), is the centrality of resonance to the integration/binding process in GRT. Resonance is not a part of IIT. Similarly, IIT’s exclusion mechanism renders all subsidiary conscious entities non-conscious and unified into a single conscious awareness. As discussed above, GRT follows the principle that the many become one and are increased by one – there is no extinction of subsidiary conscious entities.

Dehaene’s version of GWT is similar in a number of ways to our GRT, including the concepts of phase transitions; the importance of synchrony/resonance; accepting the reality of consciousness as a physical and evolutionarily useful phenomenon; viewing consciousness as a multi-layered affair; and accepting subjective reports as good data (Dehaene, 2014). A key difference between the two theories, however, is that GWT is explicitly meant to explain human consciousness and the differences between dominant consciousness, which is what we humans enjoy as normal conscious awareness, and how it relates to subconscious and preconscious processes.

We largely agree with GWT insofar as wherever Dehaene and his colleagues describe “consciousness” we would describe this as “dominant consciousness.” This is the case because GRT suggests that there are many levels of conscious awareness in humans and many other animals (and maybe even non-biological entities), but we as humans are only able to directly access dominant consciousness, which is the top of the chain in the nested hierarchy that constitutes normal human consciousness. Nested conscious entities in GRT are the subconscious and preconscious processes in GWT, from the perspective of the dominant consciousness. And each level of nested consciousness will have subsidiary conscious entities that are subconscious to it. So rather than viewing subconscious or preconscious entities as essentially zombie agents (Koch, 2004), we view these lower-level entities as conscious for themselves, but generally in a far more rudimentary manner than dominant human consciousness.

There are a number of other key differences between GRT and GWT, including: (1) GRT is a general theory that is meant to apply to all potential types of consciousness in all physical structures, not just neuronal-based consciousness or mammalian or even vertebrate consciousness; (2) GRT is panpsychist, GWT is materialist because it suggests that consciousness emerges at some point of biological complexity and is not otherwise present; (3) GRT has a quantification framework that allows the calculation of spatial and temporal boundaries and the capacity for phenomenal content in specific entities (Hunt, 2011, 2019; GWT has neither); (4) GRT is a solution to the combination problem of consciousness and the Hard Problem more generally; GWT doesn’t address these problems explicitly.

Although our proposed theory offers a possible way of accounting for how micro-conscious entities can combine to form macro-conscious entities, we acknowledge that even if resonant systems bind in the manner we are suggesting, it does not *necessarily* follow that all types of resonating structures are conscious – we could, of course, be wrong about our axioms and posited mechanisms for the combination of consciousness! Our suggested framework will need substantial empirical support before it can be considered a complete and viable theory. We argue, in general, for an approach that entertains without necessarily endorsing various approaches to the problems of consciousness.

Other conceptual frameworks are possible. Indeed, one of us has developed (Riddle and Schooler, 2019) a related theory of the organization of neural systems that posits the existence of nested information processing hierarchies [termed Nested Observer Windows (NOWs)], distributed across multiple spatial and temporal scales. In this scale-free model of cognition, biological systems at various levels ranging from neural networks to proteins, exhibit rhythmic input–output cycles and share information laterally via *coherence*, while bottom–up/top–down information-processing is orchestrated via *cross frequency coupling*. The NOW model has much in common with the shared resonance approach in the present paper in terms of coherence/resonance/sync being key for the combination of lower-order observers into higher-order ones. The NOW model is consistent with the possibility that some kind of consciousness occurs at all lower levels in neural hierarchies, no matter how rudimentary such consciousness may be (as the General Resonance Theory developed in the present paper posits). However, the NOW theory does not assume that all nested observer windows necessarily entail subjective experience. Rather, in the NOW model, consciousness may emerge at a certain level of system complexity.

Given the elusiveness of objective measures of the necessarily subjective aspects of consciousness, data suggesting the presence of consciousness at lower levels of neural complexity, for example, will never be considered incontrovertible evidence by all parties. Indeed, no incontrovertible evidence of consciousness beyond one’s own is epistemologically possible, as mentioned – this is a key point that bears repeating. Nevertheless, such findings would add to the weight of evidence in support of the views proposed in the present paper, and the key purpose of the present paper is to inspire others to propose and complete such tests for various theories of consciousness, not just for General Resonance Theory.

## Conclusion

In sum, our resonance theory of consciousness attempts to connect ongoing research efforts in various fields with the notion, suggested in some manner by various thinkers over the last two decades, that shared resonance (synchronization) is key to the nature of consciousness. More specifically, we suggest that the “easy part” of the Hard Problem – what is known generally as the combination problem – may be resolved by looking to shared resonance as a general mechanism for combination of micro-conscious entities into macro-conscious structures, without extinguishing the micro-conscious entities. This process provides a possible framework for addressing the combination of consciousness in all actual entities – that is, we posit this as a general mechanism applicable to all physical systems – not just in the context of mammalian or vertebrate consciousness. We address the various combination problems posed by Chalmers (2017) in Supplementary Appendix 2. The higher speed of information exchange made possible by various energy pathway phase transitions in biological systems allows biological life to achieve larger-scale resonant structures than would otherwise be possible – and thus significantly larger macro-conscious entities than are achieved in non-living systems.

## Author Contributions

TH wrote most of the draft and incorporated feedback through numerous rounds of revisions and discussion from JS.

## Conflict of Interest

The authors declare that the research was conducted in the absence of any commercial or financial relationships that could be construed as a potential conflict of interest.

## Footnotes

1 The two authors differ somewhat in their commitment to panpsychism. Hunt views panpsychism as the most compelling account of the relationship between consciousness and the physical world. Schooler leans toward panpsychism but is also open to alternative views. In particular, he is sympathetic to the view that consciousness may not reside in the lowest possible elements of matter, but rather may emerge at some level in the hierarchy of nested information processing systems. Although the two authors differ in their confidence in panpsychism, they share the view that shared resonance likely plays an important role in binding distinct information processing modules into unified experiential states.

2 “Micro-conscious entity” or “micro-consciousness” is defined loosely as a type of consciousness that is either the lowest level of consciousness possible, or close thereto. It is not meant to be a precise term; rather, it is meant to be an aid for distinguishing more complex forms of consciousness, like what we expect to occur in mammals and other vertebrates (and possibly further down the chain of biological complexity) and the postulated highly rudimentary states of consciousness present in molecules and atoms.

3 Real talk: Everything is made of fields (n.d.). Retrieved from https://www.symmetrymagazine.org/article/july-2013/real-talk-everything-is-made-of-fields.

4 Although sympathetic to the notion that fireflies are conscious and have intentions, Schooler finds Strogatz’s approach, described below, more convincing than positing that fireflies deliberately synchronize their flashing.

5 Chalmers (1996, p. 213) makes these requirements more explicit in Chalmers (1997): “To have a fundamental theory that we can truly assess, we will need a fundamental theory with details. That is, we will need specific proposals about psychophysical laws, and specific proposals about how these laws combine, if necessary, so that ultimately we will be able to (1) take the physical facts about a given system, (2) apply the psychophysical theory to these facts, and thus (3) derive a precise characterization of the associated experiences that the theory predicts.” Chalmers (1997), re-printed in Shear et al. (2000, p. 420).
