# Erratum: The Easy Part of the Hard Problem: A Resonance Theory of Consciousness

**DOI:** 10.3389/fnhum.2020.596409

**Published:** 2020-09-04

**Authors:** Frontiers Production Office

**Affiliations:** Frontiers Media SA, Lausanne, Switzerland

**Keywords:** consciousness, Hard Problem of consciousness, resonance, self-organization, coherence

Due to a production error, block quotes were formatted as normal text throughout the article.

The publisher apologizes for this mistake. The original article has been updated.

