# Supplementary material for: The Easy Part of the Hard Problem: A Resonance Theory of Consciousness
Source: Front Hum Neurosci. 2019 Oct 31;13:378. doi: 10.3389/fnhum.2019.00378 (PMC6834646; doi:10.3389/fnhum.2019.00378)
Supplement: Supplementary file 1 [file Data_Sheet_1.docx]

***Appendix 1: Summary of the general resonance theory of consciousness***

We address the following questions in this short summary: How do complex minds form? Why is the total greater than the sum of its parts when it comes to minds? What is the best approach for resolving the various combination problems?

What follows is a numbered summary of the general resonance theory of consciousness:

1. Resolving the hard problem and the combination problem should focus on energy/information flows and the causal structure that is part and parcel of such energy/information flows.

2. Energy/information flows are real physical structures that carry both causal impact and psyche/consciousness/mind.

3. Less complex processes have, by definition, less causal structure and thus less energy/information flows, and less consciousness.

4. Collections or aggregates of matter transcend the state of being “mere aggregates” (there is no combination of mind into a larger whole) when the constituents of such aggregates of matter enjoy a shared resonance of at least some energy/information flows.

5. All aggregates are interconnected in some manner with all other aggregates through normal physical forces, but it takes time for such connections to be made because of finite speeds for such causal connections to travel.

6. Constituents of aggregates that are in sufficient proximity for resonance-based energy/information flows to take place, within a certain timeframe, often combine and transcend the state of being “mere aggregates.”

7. The required timeframe for achieving shared resonance depends on the resonance frequency(ies) of the constituents and aggregates at issue.

8. Constituents that resonate at the same or similar frequencies in proximity will often combine into a larger consciousness (macro-consciousness) because their energy/information flows combine through coherence of their resonance frequencies, like lasers achieving coherence when photons resonate at the same frequency.

9. The velocity and frequencies of the particular resonance chains, in each case, determines the size and shape of the particular combination of resonating constituents in each moment.

10. Living organisms have, through long-term evolutionary processes, learned to take advantage of faster energy/information flows, such as electrochemical channels and electrical and magnetic fields (and possibly quantum field effects also), thus allowing physically larger resonating structures to occur.

11. As such, a living human body weighing about the same as a large boulder has far greater energy/information exchange among its constituents than does the boulder, which can rely only on thermodynamic and gravitational energy/information exchanges.

12. Accordingly, the sum of the human body’s combined consciousness is greater than its parts because of the much greater energy/information flows occurring throughout the human body, which constitute real physical structure; without these connections the human body would be a “mere aggregate” like the boulder. The sum total consciousness of the parts would be far lower without the extensive interconnections throughout the human body that are made possible by electrochemical and electromagnetic field energy/information flows.

***Appendix 2: Addressing the various types of combination problems***

Chalmers 2013 examines the combination problem and various proposed solutions. He describes not just one but three main types of combination problems: 1) the subject irreducibility problem; 2) the quality combination problem; 3) the structure combination problem.

We describe briefly how our approach addresses these three aspects of the combination problem. In summary, our resonance theory of consciousness provides essentially the same solution to each of the three combination problems: shared resonance in proximity leads to combination of entities through a relatively stable causal structure and significantly higher data bandwidths between the components of each higher-level conscious entity. The net result of achieving a shared resonance in proximity is a step change in speed and bandwidth of information flows, which, in turn, allows a unified entity to arise where before there was only a mere aggregate.

This is not, however, an all or nothing affair. Rather, resonance can be achieved momentarily, resulting in a flicker of shared consciousness, and then disappear in the next moment for various reasons. One of the defining features of biological life and biological consciousness is the ability to create *relatively stable* structures that allow for a persistent type of consciousness, flickering on and off with wakefulness and sleep, but continuing during the lifetime of the entity at issue, in terms of a store of memories, values, history, and relationships to the surrounding world. This pattern is always changing in each moment, but it retains enough relative stability to allow for continuity of consciousness over time.

*1. Subject irreducibility problem*

Chalmers asks, as the first type of combination problem: “how do microsubjects combine to yield macrosubjects?” We explained above how the oscillating nature of all actual entities allows combination by achieving a shared resonance, with the speed of the specific information flows that are present within each oscillation time period determining the size of each actual entity in each moment. Biological entities have mastered much faster types of information channels (nerves, electrical fields, etc.), through various types of resonance, allowing for much larger actual entities to form and to be sustained as semi-stable patterns over time.

*2. Quality combination problem*

Chalmers next asks “how do microqualities combine to yield macroqualities?” He adds: “Here macroqualities are specific phenomenal qualities such as phenomenal redness (what it is like to see red), phenomenal greenness, and so on. It is natural to suppose that microexperience involves microqualities, which might be primitive analogs of macroqualities. How do these combine?”

Our answer is the same as above because all actual entities include, by definition, the qualities of consciousness. Their combination, as described above, includes the combination of their qualities into a macro-conscious subject, with the experienced qualities of said subject being the sum of all included sub-entities. Just as a musical note or chord is the sum of its constituents, or a paint color mixed from other colors is the sum of its constituents, the qualities of each macro-conscious entity are the sum of its constituents.

The related “palette problem” (Goff 2017) is resolved under this approach by recognizing that even if the fundamental qualia in micro-conscious entities are limited (which we don't take a position on), the nested hierarchy that constitutes any mammalian consciousness, or similar level of biological complexity, includes, due to its complexity, more complex qualia in exactly the same manner as animal behavioral complexity increases with size and neural development. Each level of organizational and structural complexity adds the capacity for additional qualia complexity, and the dominant consciousness that we call waking consciousness includes every lower level in some manner, even if isn’t (as is generally the case) directly accessible by the dominant waking consciousness.

*3. Structure combination problem*

Chalmers asks: “how does microexperiential structure (and microphysical structure) combine to yield macroexperiential structure?” He adds: “Our macroexperience has a rich structure, involving the complex spatial structure of visual and auditory fields, a division into many different modalities, and so on. How can the structure in microexperience and microstructure yield this rich structure?”

Our response is again the same: the physical structures that support consciousness (they are two sides of the same coin, so we could as well state instead “the experiential structures that support physical structures”) are the product of (nested) resonance at various levels. All actual entities are conscious/experiential to some degree, but the fundamental physical components like electrons, protons, etc., are minimally conscious. These complex structures are a hierarchy of nested resonant structures. It is likely that consciousness only becomes non-negligible in complex structures like animal bodies/brains (but we can speculate about larger-scale but far slower consciousness in stars, galaxies, etc., depending on the stability of internal structures to those entities). The physical structures of animal bodies/brains are the substrate for information/causal pathways that lead to the combination of consciousness. As described above, the speed of information flows is the limiting factor for the size of each macro-consciousness.

The similarity of these answers is a benefit rather than a detriment, highlighting the simplicity and coherence of our conceptual structure.
